# Effects of galactosyltransferase on EPS biosynthesis and freeze-drying resistance of *Lactobacillus acidophilus* NCFM

**DOI:** 10.1016/j.fochms.2022.100145

**Published:** 2022-11-14

**Authors:** Lingyu Kong, Yuze Huang, Xiaoqun Zeng, Congyan Ye, Zhen Wu, Yuxing Guo, Daodong Pan

**Affiliations:** a State Key Laboratory for Managing Biotic and Chemical Threats to the Quality and Safety of Agro-products, Ningbo 315211, China; b Key Laboratory of Animal Protein Food Processing Technology of Zhejiang Province, College of Food and Pharmaceutical Sciences, Ningbo University, Ningbo 315800, China; c School of Food Science and Pharmaceutical Engineering, Nanjing Normal University, Nanjing 210097, China

**Keywords:** EPS, exopolysaccharideS, LAB, lactic acid bacteria, GalT, galactosyltransferase, NCBI, National Center for Biotechnology Information GenBank, MRS, de Man, Rogosa and Sharpe, LB, Luria-Bertani, PCR, polymerase chain reaction, ELISA, enzyme linked immunosorbent assay, RT-qPCR, real-time quantitative polymerase chain reaction, FT-IR, Fourier transform infrared spectroscopy, GO, gene ontology, DEG, differentially expressed gene, KEGG, Kyoto Encyclopedia of Genes and Genomes, MF, molecular function, BP, biological process, CC, cellular component, PTS, phosphotransferase system, PEP, phosphoenolpyruvate, QS, quorum sensing, Overexpression, Galactosyltransferase, Freeze-drying, Exopolysaccharide

## Abstract

Galactosyltransferase (GalT) is an important enzyme in synthesizing exopolysaccharide (EPS), the major polymer of biofilms protecting cells from severe conditions. However, the contribution to, and regulatory mechanism of GalT, in stressor resistance are still unclear. Herein, we successfully overexpressed GalT in *Lactobacillus acidophilus* NCFM by genetic engineering. The GalT activity and freeze-drying survival rate of the recombinant strain were significantly enhanced. The EPS yield also increased by 17.8%, indicating a positive relationship between freeze-drying resistance and EPS. RNA-Seq revealed that GalT could regulate the flux of the membrane transport system, pivotal sugar-related metabolic pathways, and promote quorum sensing to facilitate EPS biosynthesis, which enhanced freeze-drying resistance. The findings concretely prove that the mechanism of GalT regulating EPS biosynthesis plays an important role in protecting lactic acid bacteria from freeze-drying stress.

## Highlights

• *epsF* gene encoding GalT was overexpressed in *Lactobacillus acidophilus*.
• Overexpressed GalT increased EPS yield and lyophilized survival rate of *L. acidophilus*.
• GalT mainly affected carbohydrate metabolism, PTS, and QS of cells.
• The main affected metabolic pathways were to promote the production of EPS.
• EPS biosynthesis was positively correlated with freeze-drying resistance.

## 1 Introduction

Exopolysaccharide (EPS) is the main component of biofilms and is essential for resistance to extreme environments (Zannini, Waters, Coffey, & Arendt, 2015). Thermophilic, psychrophilic, and acidophilic bacteria produce abundant EPS and grow within biofilms (Yin, Wang, Liu, & He, 2019). EPS-producing lactic acid bacteria (LAB) have been extensively used for food, cosmetics, and medicine applications (Korcz & Varga, 2021). EPS comprises homopolysaccharide (HoPS), which is uniformly composed of glucose or fructose, and heteropolysaccharide (HePS), which consists of two to eight different monosaccharides (Torino, Font de Valdez, & Mozzi, 2015). EPS biosynthesis of LAB is impacted by several variables, including growth phase, pH, and medium composition (Patel, Majumder, & Goyal, 2011). These have been reported that LAB producing EPS will have significant differences in preferences for different carbon sources due to their characteristics (Yuliana, Sumardi, Jatmiko, Rosaline, & Iqbal, 2020), and Ca^2+^ is a vital factor in improving EPS yield by up-regulating EPS synthesis-related gene expression and main metabolic pathways that provide substrates (Jiang, Zhang, Zhang, Zulewska, & Yang, 2022). Low temperature can also promote EPS production in a certain temperature range (Esteban, Pessione, Fontana, Mazzoli, & Pessione, 2016). LAB EPS biosynthesis is governed by the *eps*/*cps* cluster containing approximately 13–23 open reading frames with multiple functions, including regulation, synthesis of repeating units, polymerization of repeating units, chain length determination, and export (Zhou, Cui, & Qu, 2019). For example, *Streptococcus thermophilus* S-3 harbors 13 *eps* genes encoding EPS biosynthesis-related proteins, including regulatory proteins (EpsAB) for *eps* operons, polysaccharide length-regulated proteins (EpsCD), glycosyltransferases for the synthesis of repeating units (EpsEFGHI), and proteins for polymerization and secretion (EpsJKLM) (Xiong et al., 2019). *Lactobacillus acidophilus* is an application-oriented probiotic with important characteristics that include regulating intestinal flora, improving immunity, and treating digestive disorders (Troche et al., 2020). *L. acidophilus* also has a functional 14-gene area of EPS biosynthesis (Zheng et al., 2021). Among these proteins, glycosyltransferases, such as glucosyltransferase, galactosyltransferases (GalT), and rhamnosyltransferase, are the key enzymes that catalyze the transfer of sugar moieties from activated donor molecules (UDP-glucose, UDP-galactose, dTDP-rhamnose, etc.) to specific lipophilic acceptors, forming glycosidic bonds to synthesize a variety of repeating units (Soumya & Nampoothiri, 2021) to compose diverse EPS. Notably, van Kranenburg et al. (1999) analyzed the composition of EPS of 16 LAB, and galactose was present in all 16, suggesting the universality and importance of GalT in LAB.

The freeze-drying technique is commonly used in processing bacterial powders, which can effectively ensure the stability and efficiency of probiotic preparations and starters, as well as the preservation of strains. However, freeze-drying can cause irreparable damage to cells. The first step in the lyophilization process is freezing the samples. The freezing damages to cells are mainly due to the freezing rate. Excessive freezing rate will generate intracellular ice crystals, resulting in cell death due to the damage of organelles and cell membranes, or slow formation of extracellular ice crystals caused by slow freezing rate will gradually form hypertonic solution outside the cells, leading to cell dehydration, shrinkage, and inactivation (Rockinger, Funk, & Winter, 2021). After freezing, the water of the frozen sample will be removed by sublimation in a low-temperature vacuum environment. The main damage during the drying process is the loss of binding water that assists in the arrangement of phospholipids and stabilizes biological macromolecules, resulting in reduced fluidity, functionality, and integrity of cell membranes (Chen, Chen, Li, & Shu, 2015). Polysaccharides, especially natural polysaccharides, are reportedly reliable as freeze-drying protectants (Li et al., 2019). And it was also that cell viability would be increased to varying degrees when cells were exposed to stresses. Our prior work showed that the glycosyltransferase activity and polysaccharide content of *L. acidophilus* significantly rose while its freeze-drying survival rate was increased with heat shock (Zhen et al., 2020), which implies that extreme stress can stimulate the expression of glycosyltransferases.

We hypothesize that glycosyltransferases, especially GalT, have a potential regulatory mechanism between EPS biosynthesis and freeze-drying resistance. To explore this mechanism, *epsF* (National Center for Biotechnology Information GenBank (NCBI) accession No. CP000033.3: *LBA1732,* https://www.ncbi.nlm.nih.gov/nuccore/CP000033.3), which encodes GalT, was cloned and overexpressed in *L. acidophilus* NCFM to evaluate the influence of the changes in GalT activity on EPS biosynthesis and freeze-drying survival rate. RNA-Seq was used to further investigate the mechanism of how *epsF* affects EPS biosynthesis and the interaction between GalT and other proteins. This study aims to reveal the regulatory effect of GalT between EPS biosynthesis and freeze-drying resistance of lactic acid bacteria and provide a basis for the construction of LAB with high EPS yield and stress resistance.

## 2 Materials and methods

### 2.1 Chemicals, enzymes, and bacterial growth conditions

Glucose, glycine, glycerol, sucrose, sodium chloride, and erythromycin were purchased from BBI Co., Ltd. (Shanghai, China). *Sac* Ⅰ and *Hind* III restriction enzymes were purchased from TaKaRa Bio Inc. (Shiga, Japan). *L. acidophilus* NCFM purchased from Guangdong Microbial Culture Collection Center (Guangdong, China) was cultured in de Man, Rogosa, and Sharpe (MRS) medium (Hopebio, Qingdao, China) at 37 °C. *Trans*1-T1 (a cloning competent *Escherichia coli*) purchased from TransGen Biotech Co., Ltd. (Beijing, China) and *E. coli* DH5ɑ (carrying pMG36e) maintained in our laboratory (Ningbo University, China) were cultured in Luria-Bertani (LB) medium (Hopebio, Qingdao, China) at 37 °C with a shaking rate of 200 rpm. Plasmid pMG36e was used as an expression vector.

### 2.2 Cloning of *epsF*

Genomic DNA from *L. acidophilus* NCFM was extracted using *EasyPure*® Bacteria Genomic DNA Kit (TransGen Biotech, Beijing, China), as recommended in the user manual. *epsF* was amplified (primers *epsF*-F/R in Table 1) from genomic DNA by polymerase chain reaction (PCR) using T100™ Thermal Cycler (Bio-Rad, Hercules, CA, USA). The PCR procedure was: initial denaturation at 95 °C for 3 min; 30 cycles of denaturation at 95 °C for 15 s, annealing at 60 °C for 15 s, and extension at 72 °C for 45 s; and final extension at 72 °C for 5 min. The purified PCR product of *epsF* was sequenced by Sangon Biotech Co., Ltd. (Shanghai, China) to confirm its correctness for the next step.

**Table 1:** Primers used in this study

No.	Primer	Sequence (5′–3′)	Restriction enzyme
1	*epsF*-F	AAAAATTCGTAATTC*GAGCTC*ATGAAAATAAAAATTTTAGTTGCTGCA	*Sac* Ⅰ
2	*epsF*-R	GTTTTCAGACTTTGC*AAGCTT*TTAAAAGTGTGTTTTAGTCTTTATTCCCA	*Hind* III
3	pMG36e-F	GTCGATCGAATTCGGTCCTCGGGAT	
4	pMG36e-R	GAAGTCAGCTGCCTAAGCAAGGTTC	
5	*epsF*-T-F	GGTCTTGTCCACTATCGTCG	
6	*epsF*-T-R	GGCAACTACATCTCTCGTTCT	
7	16S rRNA-F	CACCGCTACACATGGAG	
8	16S rRNA-R	AGCAGTAGGGAATCTTCCA	

The italics in the sequence denote the restriction enzyme sites.

### 2.3 Construction of recombinant plasmid

pMG36e was extracted by using SanPrep Column Plasmid Mini-Preps Kit (Sangon Biotech, Shanghai, China) from *E. coli* DH5ɑ, and digested with *Sac* Ⅰ and *Hind* III. *epsF* was inserted into linear pMG36e by recombinant enzyme-seamless cloning method using ClonExpress® Ⅱ One Step Cloning Kit (Vazyme, Nanjing, China) to construct the recombinant plasmid pMG36e-*epsF*. The ligation mixtures were transferred into *Trans*1-T1 competent cells by heat shock at 42  °C after being placed in an ice bath for 30 min to generate the stable recombinant plasmids. The recombinant *Trans*1-T1 cells were coated on LB agar medium with 200 μg/mL erythromycin to screen the positive clones identified by colony PCR (primers *epsF*-F/R). The PCR products were sequenced by Sangon Biotech Co., Ltd. (Shanghai, China) to confirm their accuracy.

### 2.4 Transformation of *L. acidophilus* NCFM

#### 2.4.1 Preparation of *L. acidophilus* NCFM competent cells

*L. acidophilus* competent cells were prepared following a modified method of Song (Song, Xiong, Kong, Wang, & Ai, 2018). Overnight 2 % (v/v) activated *L. acidophilus* NCFM was inoculated in MRS broth supplemented with 0.5 % glucose and 0.5 % glycine, and cultured to exponential growth phase (optical density at 600 nm - OD_600_ ≈ 0.6) detected by Tecan Infinite 200 Pro (Tecan, Männedorf, Switzerland). The bacteria obtained by centrifugation (220×*g*, 10 min) were washed twice with prechilled sterile deionized water and then twice with prechilled solution A (10 % glycerol + 10 % sucrose). Finally, cells were resuspended in a onefold volume of solution A. The competent cells were immediately snap-frozen in liquid nitrogen and stored at −80 °C.

#### 2.4.2 Electrotransformation

Electrotransformation was used to transform pMG36e-*epsF* into *L. acidophilus* NCFM. The mixture containing about 1 μg pMG36e-*epsF* and 100 μL freshly prepared competent cells was electroporated at 1.2 kV for 4 ms by MicroPulser Electroporator 1652100 (Bio-Rad, Hercules, CA, USA). After electroporation, the mixture was immediately transferred to 1 mL prechilled MRS broth and incubated at 37 °C for 3 h. The *L. acidophilus*-*epsF* recombinant strain was initially screened on MRS agar medium containing 6 μg/mL erythromycin and identified by colony PCR (primers pMG36e-F and *epsF*-R, Table 1). pMG36e was transformed into *L. acidophilus* NCFM to similarly obtain the control strain *L. acidophilus*-0.

### 2.5 Determination of GalT activity

The difference in GalT activity between *L. acidophilus*-0 and *L. acidophilus*-*epsF* was determined by enzyme linked immunosorbent assay (ELISA) according to the instructions of the Microorganism Galactosyltransferase (GALT) ELISA Kit (K-X Biotechnology, Shanghai, China) to determine GalT activity. The two strains were grown in 100 mL MRS broth until the exponential growth phase and harvested by centrifugation at 885×*g* for 5 min. The precipitates were washed three times with PBS and resuspended in 20 mL PBS. The suspension was ultrasonicated on ice (400 W, 3 s pulse, 10 s pause; 80 cycles) to disrupt the cells. The lysate was centrifuged at 1600×*g* for 10 min at 4 °C. The supernatant was examined using the kit to determine its GalT activity.

### 2.6 Real-time quantitative PCR (RT-qPCR)

Total RNA from *L. acidophilus*-0 and *L. acidophilus*-*epsF* was separately extracted using HiPure Bacterial RNA Kit (Magen, Guangzhou, China). cDNA was reverse-transcribed from equal quality (≤1μg) of the total RNA for RT-qPCR using TransScript® All-in-One First-Strand cDNA Synthesis SuperMix (TransGen Biotech, Beijing, China) following the manufacturer’s instructions.

RT-qPCR using TransStart® Tip Green qPCR SuperMix (TransGen Biotech, Beijing, China) and LightCycler® 96 (Roche, Basel, Switzerland) involved initial denaturation at 94 °C for 30 s and 45 cycles of denaturation at 94 °C for 5 s, annealing at 60 °C for 30 s, and extension at 72 °C for 30 s. 16S rRNA was the internal reference (Kim et al., 2015) for *epsF*. Primers No. 5–8 (Table 1) were designed for this step. The gene expression levels were estimated using the $2^{-\Delta \Delta Ct}$ method (Livak & Schmittgen, 2001). Each experimental group was repeated three times.

### 2.7 Determination of freeze-drying survival rate

Overnight 2 % (v/v) activated *L. acidophilus*-0 and *L. acidophilus*-*epsF* were separately inoculated in 100 mL MRS broth at 37 °C for 8 h. The OD_600_ was adjusted to the same value, and each culture was divided into two 50-mL portions. One portion was centrifuged (4 °C, 885×*g*, 10 min). The other was frozen at −80 °C. Cells from the first portion were washed three times with 0.9 % sterile normal saline before being suspended in 500 μL normal saline. The bacterial suspension was diluted (10^−2^, 10^−3^, 10^−4^, 10^−5^, 10^−6^, 10^−7^, 10^−8^), spread on MRS agar, and cultured at 37 °C for 24 h to count the number of viable bacteria (E et al. 2020). The frozen broth was vacuum freeze-dried (-49 °C, 24 h, 9 Pa), suspended in sterile normal saline to the initial OD_600_ value, and treated as just described. The freeze-drying survival rate is calculated as follows:

$$\text{X}=\left({\text{N}}_{\text{fd}}/{\text{N}}_{0}\right)\times 100\%$$

where X is the freeze-drying survival rate, N_0_ is the number of colonies in the non-lyophilized treatment group, and N_fd_ is the number of colonies in the lyophilized treatment group.

### 2.8 EPS determination

#### 2.8.1 Extraction and purification of EPS

Ethanol precipitation of EPS was performed as previously described (Li, Mutuvulla, Chen, Jiang, & Dong, 2012). Briefly, *L. acidophilus*-0 and *L. acidophilus*-*epsF* were cultured in MRS broth containing 6 μg/mL erythromycin to OD_600_ ≈ 0.5. The fermentation supernatants were harvested by centrifugation (4 °C, 615 × g, 20 min). Each supernatant was heated at 100 °C for 15 min and then cooled to room temperature before adding 17 % (w/v) 85 % trichloroacetic acid. Each sample was stored at 4 °C for 8 h to remove proteins. Three volumes of prechilled anhydrous ethanol were added to the supernatant, which was stirred at 4 °C overnight to precipitate EPS. The EPS was dissolved in ultrapure water (60 °C) and dialyzed (8000 Da) at 4 °C for 72 h (water replaced every 4 h). The EPS solution was freeze-dried.

#### 2.8.2 Concentration and structure of EPS

EPS concentration was determined by the phenol–sulfuric acid method (Wang et al., 2017). A series of concentration gradients (0, 20 %, 40 %, 60 %, 80 %, 100 %, v/v) of 1 mL glucose were prepared as standards. Each glucose solution was mixed with 0.5 mL of 6 % phenol and 2.5 mL of concentrated sulfuric acid and chilled on ice for 30 min. The OD_490_ of each sample was measured and an OD_490_ standard concentration curve was plotted. The freeze-dried EPS was subsequently used to prepare 0.1 mg/mL samples that were treated as described above to calculate the EPS concentration using the OD_490_ concentration standard curve.

### 2.9 RNA-Seq analysis

Total RNA was extracted in the same way as RT-qPCR from *L. acidophilus*-0 and *L. acidophilus*-*epsF*. mRNA was purified from total RNA using Ribo-off rRNA Depletion Kit (Vazyme). A cDNA library was constructed and sequenced by Illumina Hiseq 4000 (Illumina, San Diego, CA, USA). The expression levels of genes were estimated by the expected number of fragments per kilobase of the transcript sequence per million base pairs sequenced. Differentially expressed genes (DEGs) between the two groups were identified based on the two screening conditions of differential expression fold (log2 (Fold Change) > 1) and significance (*P* < 0.05). The biological functions of DEGs were annotated by gene ontology (GO) enrichment analysis. Significantly enriched GO terms were screened by topGO. The Kyoto Encyclopedia of Genes and Genomes (KEGG) database (https://www.genome.jp/kegg/pathway.html) was used to retrieve pathways with significant enrichment of DEGs.

### 2.10 Statistical analysis

All treatments were conducted in three replicates, and data were shown as mean values ± standard deviation (SD) using the SPSS statistical software (IBM, Chicago, IL, USA). Independent sample t-test was used to analyze the data between two groups of *L. acidophilus*-0 and *L. acidophilus*-*epsF* to determine if there were significant differences at *P* < 0.05, and “*” for each of the two sets of samples indicates a significant difference.

## 3 Results and discussion

### 3.1 Construction of recombinant plasmid

**Fig. 1:**
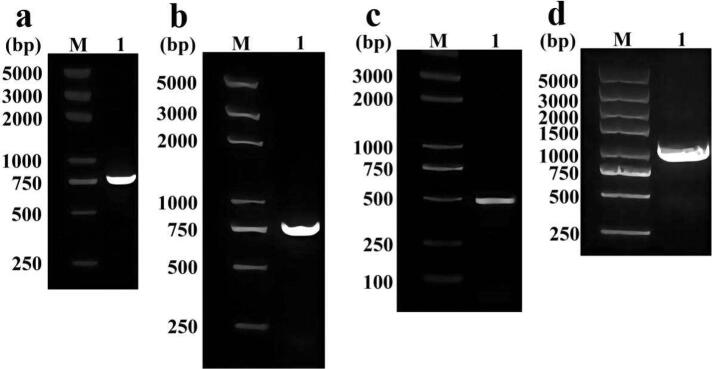
Agarose gel electrophoresis for PCR products. Lane-M: marker. Lane-1(a): identification of amplified *epsF*; Lane-1(b): identification of *epsF* in Trans1-T1 cells; Lane-1(c): identification of pMG36e in *L. acidophilus*-0; Lane-1(d): identification of pMG36e-*epsF* in *L. acidophilus*-*epsF*.

Since the PCR product of *epsF*-F/R (Fig. 1a) matched the sequence of *epsF* (774 bp) *epsF* from *L. acidophilus* NCFM in NCBI (alignment rate was 100 %), the target gene *epsF* was correctly amplified. *epsF* was then ligated with pMG36e, and it was verified that the pMG36e-*epsF* was correctly carried in *Trans*1-T1 cells by colony PCR (Fig. 1b) and sequencing.

### 3.2 Overexpression of *epsF* in *L. acidophilus* NCFM

#### 3.2.1 Construction of overexpression strain *L. acidophilus*-*epsF*

The sequence length of a fragment amplified by pMG36e-F/R on pMG36e is theoretically 479 bp, and the sequence length of a fragment (containing *epsF)* amplified by pMG36e-F/*epsF*-R on pMG36e-*epsF* is approximately 1000 bp. Their bands were accurate (Fig. 1c and d). Their sequences were confirmed by sequencing and alignment. The results indicate the successful transformation of pMG36e-*epsF* and pMG36e into *L. acidophilus* NCFM.

#### 3.2.2 Determination of GalT activity and RT-qPCR

**Fig. 2:**
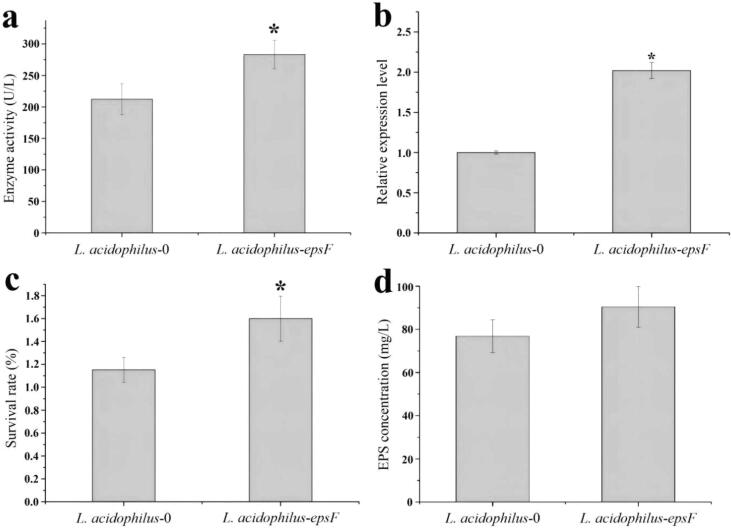
Different indicators between *L. acidophilus*-0 and *L. acidophilus*-*epsF.* (a) Enzyme activity of GalT. (b) Relative expression level of *epsF*. (c) Freeze-drying survival rate. (d)EPS concentration. * on the bar denotes a significant difference (*P* < 0.05) between the two groups. The detailed values are listed in Supplementary Table 1.

GalT activity was 33.6 % higher in *L. acidophilus-epsF* (283.7 U/L) than in *L. acidophilus*-0 (212.3 U/L) (Fig. 2a). RT-qPCR showed that the expression level of *epsF* in *L. acidophilus*-*epsF* was more than double that of *L. acidophilus*-0 (Fig. 2b). The same trend for each transformant indicated the overexpression of *epsF* in *L. acidophilus* NCFM.

### 3.3 Effects of overexpressed *epsF* on freeze-drying resistance and EPS synthesis

As indicated in Fig. 2c, the survival rate of *L. acidophilus*-*epsF* (1.60 %) was significantly higher than that of *L. acidophilus*-0 (1.15 %) (*P* < 0.05). Meanwhile, EPS concentration was increased by 17.8 % following the overexpression of *epsF* (Fig. 2d). There is a positive correlation between the increase in EPS yield and the increase in lyophilized survival rate. Similarly, Zhuang et al. (2020) found that the overexpression of gene *GSU1501* involved in EPS secretion promoted EPS biosynthesis, enhanced cell membrane stability, reduced electron transfer resistance, and increased the strain’s activity. The findings suggest that GalT may have a similar effect and preliminarily corroborates the protective role of EPS. To investigate the influence of *epsF* overexpression on the survival rate, lyoprotectants were not applied to avoid interference with the results. During freeze-drying, excessive water loss causes high osmotic pressure, imbalanced K^+^ - Na^+^ ratio, cell folding, and enzyme inactivation resulting in a low cell survival rate (Gharib, Fourmentin, Charcosset, & Greige-Gerges, 2018). Ming et al. (2009) conducted research that showed the survival rates of *Ligilactobacillus salivarius* with and without a protective agent (skim milk + sucrose) were less than 0.1 % and more than 60 %, respectively. Therefore, the survival rates were extremely low without protective measures.

Although the EPS yield of *L. acidophilus-epsF* (90.49 mg/L) is much higher than that of *L. acidophilus*-0 (76.83 mg/L), there is no significant difference (*P* > 0.05) between the two strains by statistical analysis. This situation may be due to the limited role of GalT in the cell. Similar results were reported that galactosyltransferase or rhamnosyltransferase did not significantly affect EPS yield in *Lactobacillus casei* LC2W, and dTDP-glucose 4,6 dehydratase or galactose-1-phosphate uridylyltransferase only increased EPS yield by less than 20 % (Li et al., 2015). Boels et al. (2001) overexpressed UDP-glucose pyrophosphorylase and UDP-galactose epimerase that catalyze the synthesis of UDP-glucose and UDP-galactose, respectively in *L. lactis* MG1363, which significantly increased the level of nucleotide sugar, but not EPS yield. However, Wang et al. (2016) overexpressed *epsN*, which encodes the flippase membrane protein associated with EPS output, significantly increasing EPS production by 30 % - 60 % in different LAB strains. These suggest that the correlative intracellular enzymes, including GalT, involve the synthesis of interspecific substrates for EPS, but do not significantly affect EPS output. Boels et al. (2003) carried out homologous overexpression of the entire *eps* cluster in *L. lactis* NZ9000 and successfully increased the EPS level by 4-fold, further suggesting that EPS export is limited by the overall transcription of this cluster, rather than the level of the intracellular enzymatic reaction and nucleotide sugar. Besides, as the sole carbon source for direct use in the MRS medium is glucose, with no lactose and galactose, *L. acidophilus* could not directly produce sufficient UDP-galactose for GalT catalysis, limiting the efficiency of EPS production.

### 3.4 RNA-Seq of *L. acidophilus*-0 and *L. acidophilus*-*epsF*

#### 3.4.1 Illumina sequence data

An average of 28 million raw reads for each sample were generated, with over 94 % (Q30) being usable after quality filtering (Table 2). These were aligned and mapped to the *L. acidophilus* NCFM reference genome using Tophat (https://ccb.jhu.edu/software/tophat/index.shtml). Each library had substantial proportions of reads (>98 %) mapped to the *L. acidophilus* NCFM genome (Supplementary Material Table 2), suggesting the robust quality of library construction and RNA-Seq.

#### 3.4.2 GO enrichment analysis of DEGs

**Fig. 3:**
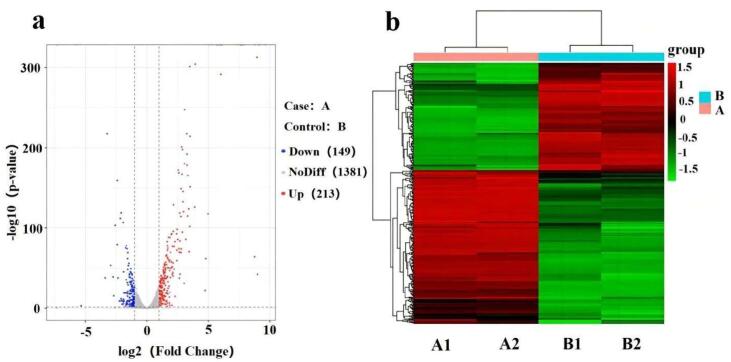
Distribution and cluster of DEGs of *L. acidophilus*-0 and *L. acidophilus*-*epsF.* A: *L. acidophilus*-*epsF*; B: *L. acidophilus*-0; (a) Volcano plot of DEGs. Red, blue, and grey dots represent up-regulated, down-regulated, and non-significant DEGs, respectively; *P* < 0.05; (b) Heat map of DEGs clustering. A1 and A2 (B1 and B2) are parallel groups. Horizontal lines above the abscissa represent genes, one sample per column. Red and blue denote high and low expression genes, respectively.

**Fig. 4:**
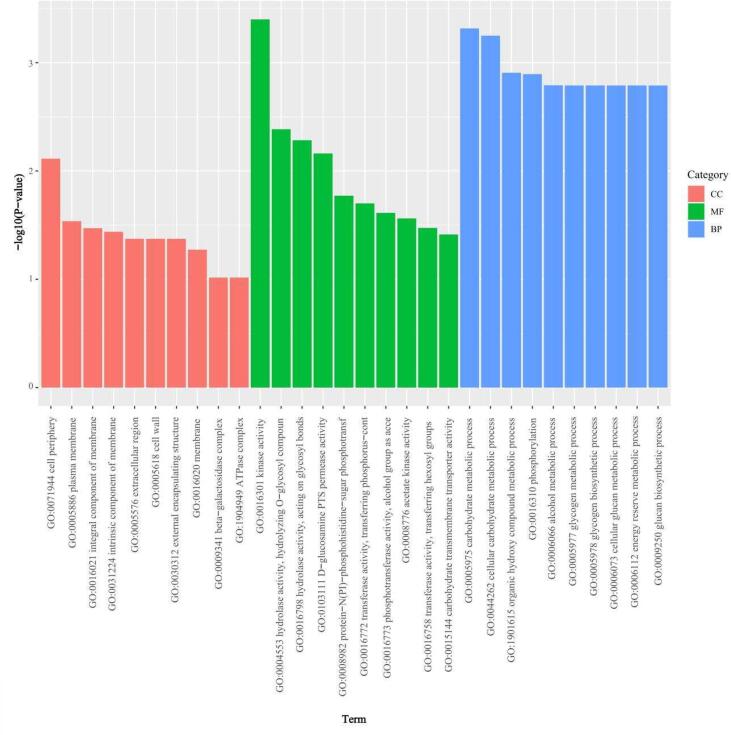
Column chart of topGO analysis of *L. acidophilus*-*epsF* compared with *L. acidophilus*-0. CC: cellular component; MF: molecular function; BP: biological process.

Between *L. acidophilus*-0 and *L. acidophilus*-*epsF*, 362 DEGs including 213 up-regulated and 149 down-regulated genes were identified (Fig. 3a). Pronounced consistency was apparent between parallel groups, and *epsF* overexpression induced alterations in overall gene transcription levels (Fig. 3b). The DEGs were categorized according to GO enrichment analysis, labeled as molecular function (MF), biological process (BP), and cellular component (CC). The top 10 significantly enriched GO terms of each category were selected (Fig. 4). CC comprised a large proportion of genes linked with “cell periphery”. MF included genes implicated in “kinase activity”, “hydrolase activity, hydrolyzing O-glycosyl compounds”, “hydrolase activity, acting on glycosyl bonds” and “ D-glucosamine PTS permease activity”. In particular, DEGs were mostly enriched in BP terms. Each BP term is linked to carbon metabolism, which is directly related to EPS biosynthesis.

#### 3.4.3 KEGG pathway analysis of DEGs

DEGs were annotated by KEGG to identify orthologous genes, illustrating the adjustments in the pathways of *L. acidophilus*-*epsF*. Raw data included 70 pathways belonging to 23 different types. The number of pathways belonging to carbohydrate metabolism and amino acid metabolism, respectively, accounted for 15.7 % and 11.4 % of the total of these pathways. DEGs involving carbohydrate metabolism were particularly prominent in KEGG pathways, especially starch and sucrose metabolism. However, few DEGs are involved in amino acid metabolism. The changes in amino acid metabolism likely mainly reflected the requirements for the synthesis of proteins (Neis, Dejong, & Rensen, 2015).

**Fig. 5:**
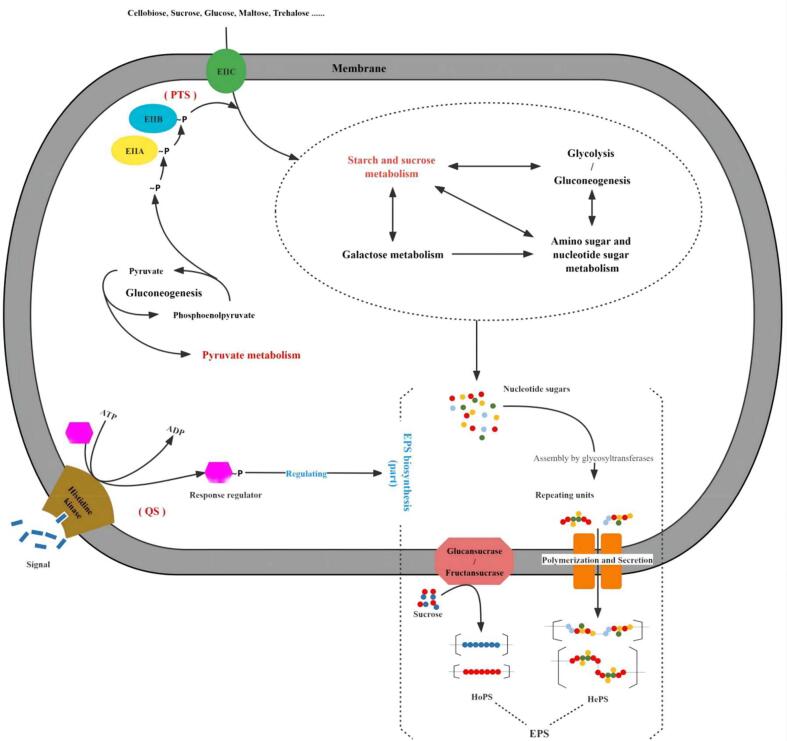
Transport and metabolism link in *L. acidophilus*-*epsF.* The pathways marked in red font show significant enrichment (*P* < 0.05). Each arrow represents the direction of material transport or the presence of mutual material exchange relationship between metabolic pathways.

Screening pathways highly relevant for EPS biosynthesis allowed an overview of the supply and demand connections between these pathways and EPS biosynthesis (Fig. 5). The phosphotransferase system (PTS) (ko02060), which is a vital membrane transport system that aids bacteria in absorbing extracellular carbohydrates for EPS biosynthesis (Galinier & Deutscher, 2017), had extremely significant variation. It displays much more up-regulated DEGs and corresponds to changes in membrane composition in CC. In this system, phosphoenolpyruvate (PEP)-protein phosphotransferase (*ptsI*, RS03335) was up-regulated, which converts phosphoenolpyruvate to pyruvate to furnish phosphoric acid groups for transporters. The accumulated pyruvate is partially converted to PEP by gluconeogenesis (ko00010) and partially removed by pyruvate metabolism (ko00620) or glycolysis (ko00010) to eliminate the metabolic burden. The up-regulated genes *celA* (RS04470), *scrA* (RS01975), and *treA* (RS05115) in PEP-PTS encode transporter components that transport extracellular monosaccharides or disaccharides like cellobiose, sucrose, and trehalose. Increased disaccharide content, such as trehalose, in cells, can enhance tolerance to freezing and drying (Vaessen, Besten, Esveld, & Schutyser, 2019). Moreover, up-regulated Crr (*exp5*, RS03170) of the EⅡA component of PEP-PTS strengthened the inward transport of maltose, glucose, and N-acetyl-D-glucosamine. The vast majority of sugars delivered by PEP-PTS are metabolically transformed by starch and sucrose metabolism (ko00500), where the expressions of β-phosphoglucomutase (*yvdM*, RS09095), ɑ-glucosidase (*agl2*, RS08835), and sucrose-6-p hydrolase (*scrB*, RS01970) were also significantly increased. “Amino sugar and nucleotide sugar metabolism” (ko00520) involves the synthesis of EPS-precursor (Padmanabhan, Tong, Wu, Lo, & Shah, 2020). Five of its six DEGs were up-regulated, including genes encoding glucose-1-P adenylyltransferase and phosphoglucomutase. Galactose metabolism (ko00052) is an important pathway for UDP-galactose synthesis (Chai, Beauregard, Vlamakis, Losick, & Kolter, 2012), but its β-galactosidase expressing genes *lacZ* (RS07175) and *lacM* (RS07200) were down-regulated, which might be greatly affected by the medium lacking lactose. Because of the lactose-free condition and the increased flux of glucose-6-phosphate, which is successively catalyzed into more UDP-glucose by phosphoglucomutase and UDP-glucose pyrophosphorylase, partial UDP-glucose would be catalyzed by UDP-glucose-4-epimerase into UDP-galactose, which is indispensable for EPS biosynthesis. Thus, even though some metabolic pathways were up-regulated, cells had to obtain the corresponding intermediates from other pathways. Finally, these up-regulated pathways did not operate at full capacity, resulting in a lower increase in EPS production than anticipated.

Quorum sensing (QS) (ko02024), a signal transduction pathway that includes extracellular signal molecules and a two-component system (including histidine kinase and response regulator), regulates biofilm formation by bacteria (Kareb & Aïder, 2019). We observed that the gene level of the two-component system (RS08805, RS08800) was up-regulated, which has a positive regulatory effect on the expression of glycosyltransferases (Senadheera & Cvitkovitch, 2008). Thus the increase of EPS yield of recombinant *L. acidophilus* in this study indicated that the formation of biofilm was promoted, which enhanced the ability of the host to resist freeze-drying stress. In addition to creating a protective barrier, a biofilm can more effectively capture nutrients from extreme environments (Yin, Wang, Liu, & He, 2019). In general, bacteria attach to a surface and then produce extracellular polymers to form biofilms (Hooshdar, Kermanshahi, Ghadam, & Khosravi-Darani, 2020). However, strains used in EPS determination were typically cultivated in liquid, where the QS signal molecules have a higher diffusivity than solid support. As a result, the signal level does not reach the recognition threshold of histidine kinase, which normally initiates QS to regulate EPS biosynthesis. This is also one of the reasons why *L. acidophilus* was unable to produce more substantial amounts of EPS.

These findings support the view that GalT can promote the uptake and metabolism of carbohydrates in *L. acidophilus*, and can regulate the reception and response of signals for QS. These changes caused by GalT regulation in the overall pathway favor EPS biosynthesis. However, *ywqD* (RS08510) encoding an EPS biosynthesis protein was down-regulated, contrary to our observation of the increased EPS yield. As well, *ytgP* (RS07905) expressing a polysaccharide-transporter was up-regulated. We suspect that the overexpressed GalT can also trigger the regulatory region in *eps* cluster to negatively regulate the synthetic regions to a certain extent in response to the abnormal expression of GalT.

## 4 Conclusion

LAB EPS and freeze-drying are important contributors to biological products. The present data reveal the regulatory mechanism of GalT between EPS biosynthesis and freeze-drying resistance in *L. acidophilus*. The GalT-overexpressed strain *L. acidophilus*-*epsF* had a higher GalT activity and a higher survival rate than the control strain. RNA-Seq findings suggested that GalT can affect PTS, carbohydrate metabolism, QS, and biofilm formation. All these are associated with EPS production. Additionally, it is possible to deduce that EPS biosynthesis and cell freeze-drying resistance are positively associated. However, the effects of the overexpressed GalT were attenuated by the limited culture conditions and self-regulation of the *eps* cluster. But these issues are solvable. It is effective to apply the more appropriate carbon sources like lactose, sucrose, or composite disaccharide, optimize the fermentation conditions (temperature, pH, carbon-nitrogen ratio, etc.) to improve the synthesis efficiency and yield of EPS and modify LAB to control the positive regulation of the *eps* cluster in the production of EPS through genetic engineering. Even so, *L. acidophilus*-*epsF* still had a greater increase in EPS yield. The collective findings indicate that GalT can promote cellular carbon flux by regulating sugar metabolism and related pathways, thereby promoting EPS biosynthesis. Abundant EPS protects cells from freeze-drying stress. The data concerning GalT and its regulatory metabolic pathways will inform future research on the transformation of LAB or the improvement of cultivation strategies to enhance EPS production by LAB, which will reduce mortality of LAB in bacteria preparations with special processing, such as freeze-drying.

## CRediT authorship contribution statement

**Lingyu Kong:** Conceptualization, Data curation, Formal analysis, Writing – original draft, Writing – review & editing. **Yuze Huang:** Formal analysis, Software, Writing – review & editing. **Xiaoqun Zeng:** Conceptualization, Supervision, Funding acquisition, Project administration, Writing – review & editing. **Congyan Ye:** Validation, Data curation, Writing – review & editing. **Zhen Wu:** Methodology, Supervision. **Yuxing Guo:** Methodology, Resources, Supervision. **Daodong Pan:** Methodology, Resources, Supervision, Project administration.

## Declaration of Competing Interest

The authors declare that they have no known competing financial interests or personal relationships that could have appeared to influence the work reported in this paper.

## Data Availability

Data will be made available on request.
