# Supplementary material for: Effects of galactosyltransferase on EPS biosynthesis and freeze-drying resistance of Lactobacillus acidophilus NCFM
Source: Food Chem (Oxf). 2022 Nov 14;5:100145. doi: 10.1016/j.fochms.2022.100145 (PMC9789326; doi:10.1016/j.fochms.2022.100145)
Supplement: Supplementary data 1 [file mmc1.doc]

**Supplementary Table 1** Statistical results of research data in Fig 2

| Strain | GalT activity (U/L) | qPCR-Relative expression level | Survival rate (%) | EPS concentration (mg/L) |
| --- | --- | --- | --- | --- |
| *L. acidophilus*-0 | 212.33 ± 11.44 | 1 ± 0 | 1.15 ± 0.11 | 76.83 ± 6.46 |
| *L. acidophilus*-*epsF* | 283.67 ± 13.60* | 2.02 ± 0.13* | 1.60 ± 0.20* | 90.49 ± 8.16 |

In the second column of qPCR data, the calculated value of *L. acidophilus*-0 as the control group was fixed at 1 without standard deviation due to the calculation method of relative values. “ * ” indicates significant difference (*P* < 0.05) between data in each column.

**Supplementary Table 2** Statistical results of cDNA library data

| Strain | Group | Reads No. | Bases (bp) | Clean reads (%) | Clean Q30 (%) | Total Mapped Reads (%) |
| --- | --- | --- | --- | --- | --- | --- |
| *L. acidophilus*-0 | 1 | 25934554 | 3890183100 | 95.14 | 95.14 | 99.10 |
|  | 2 | 30266482 | 4539972300 | 95.17 | 95.17 | 99.06 |
| *L. acidophilus*-*epsF* | 1 | 28896494 | 4334474100 | 94.52 | 94.52 | 98.98 |
|  | 2 | 28966016 | 4344902400 | 94.80 | 94.80 | 98.96 |

Reads No.: the number of sequences; Q30: the total number of bases with a base recognition accuracy more than 99.9%; Clean reads and clean Q30: high quality reads and Q30 after data filtering.
